# The potential of adoptive transfer of γ9δ2 T cells to enhance blinatumomab’s antitumor activity against B-cell malignancy

**DOI:** 10.1038/s41598-021-91784-1

**Published:** 2021-06-11

**Authors:** Yun-Hsiang Chen, Yun Wang, Cheng-Hao Liao, Shu-Ching Hsu

**Affiliations:** 1 Department of Life Science, Fu-Jen Catholic University, New Taipei City, Taiwan; 2 Center for Neuropsychiatric Research, National Health Research Institutes, Zhunan, Taiwan; 3 Manysmart Therapeutics Inc., Taipei, Taiwan; 4 Institute of Infectious Diseases and Vaccinology, National Health Research Institutes, Zhunan, Taiwan; 5 Graduate Institute of Medicine, College of Medicine, Kaohsiung Medical University, Kaohsiung City, Taiwan; 6 PhD Program in Tissue Engineering and Regenerative Medicine, National Chung Hsing University, Taichung City, Taiwan; 7 Graduate Institute of Biomedical Science, China Medical University, Taichung City, Taiwan; 8 Immunology Research and Development Center, China Medical University, Taichung City, Taiwan; 9 Department of Life Sciences, Tzu Chi University, Hualien, Taiwan

**Keywords:** Cancer, Immunology, Medical research

## Abstract

Blinatumomab, a bispecific T cell engager (BiTE) antibody targeting CD19 and CD3ε, can redirect T cells toward CD19-positive tumor cells and has been approved to treat relapsed/refractory B-cell acute lymphoblastic leukemia (R/R B-ALL). However, chemotherapeutic regimens can severely reduce T cells’ number and cytotoxic function, leading to an inadequate response to blinatumomab treatment in patients. In addition, it was reported that a substantial portion of R/R B-ALL patients failing blinatumomab treatment had the extramedullary disease, indicating the poor ability of blinatumomab in treating extramedullary disease. In this study, we investigated whether the adoptive transfer of ex vivo expanded γ9δ2 T cells could act as the effector of blinatumomab to enhance blinatumomab’s antitumor activity against B-cell malignancies in vivo. Repeated infusion of blinatumomab and human γ9δ2 T cells led to more prolonged survival than that of blinatumomab or human γ9δ2 T cells alone in the mice xenografted with Raji cells. Furthermore, adoptive transfer of γ9δ2 T cells reduced tumor mass outside the bone marrow, indicating the potential of γ9δ2 T cells to eradicate the extramedullary disease. Our results suggest that the addition of γ9δ2 T cells to the blinatumomab treatment regimens could be an effective approach to enhancing blinatumomab’s therapeutic efficacy. The concept of this strategy may also be applied to other antigen-specific BiTE therapies for other malignancies.

## Introduction

Acute lymphoblastic leukemia (ALL) is hematological cancer characterized by the rapid proliferation of large numbers of immature lymphocytes in the peripheral blood and bone marrow. The excessive immature lymphocytes can interfere with normal hematopoiesis in the bone marrow and often invade other organs such as the brain, liver, lymph nodes, and spleen. ALL represents about 25% of cancers in children but less than 1% of cancers in adults; the estimated occurrence rate of ALL is 1.7 in 100,000 persons per year in the US^1^. Based on the pathological examination, immunophenotyping, and cytogenetic analysis, ALL has been classified into different subtypes, including B- or T-cell lineage, positive or negative with Philadelphia chromosome, and leukemic cells with various molecular markers^2^. B-cell ALL is the most common subtype accounting for nearly 80% of all leukemias. Current chemotherapy regimens have improved the survival rate to 80–90% in children with B-cell ALL^3^. However, although the initial complete response rate is 80–90% in adult patients, most of the patients will relapse with resistance to chemotherapy, and the survival rate is only 40–50%. In the relapsed/refractory B-cell ALL (R/R B-ALL) patients, the prognosis is poor, and the survival rate declines to 15–50% for children and only 10% for adults^4–6^. Thus, the development of novel treatment strategies to improve outcomes in R/R B-ALL patients is desperately needed.

Bispecific T-cell engagers (BiTEs) are a novel class of fusion protein (55–60 kDa) consisting of two single-chain variable fragments (scFvs) derived from two distinct monoclonal antibodies specific to the surface antigen of tumor cells and the CD3ε subunit on all types of T cells^7^. Both scFvs are connected by a short and flexible linker that allows BiTE antibodies to draw tumor cells and T cells close to form an immunological synapse^8^. Only upon binding to the target cells, BiTEs activate cytotoxic T cells, but not naive T cells, to release perforin and granzyme B without the requirement of T cell receptor specificity and costimulatory signals, finally causing apoptosis and death of the targeted tumor cells^9^. Blinatumomab (CD19BiTE) is the first BiTE antibody approved by the US Food and Drug Administration (FDA) and the European Medicines Agency for the treatment of R/R B-ALL^10^. Blinatumomab targets CD19, a transmembrane glycoprotein expressed on normal and most neoplastic B cells but absent on hematopoietic stem cells and plasma cells^11^. Furthermore, CD19 can modulate protein tyrosine kinases and amplify PI3K signaling for cell survival and resistance to chemotherapy in B-cell malignancies^12^, making it a prominent target for BiTE.

Notably, the T cells activated by blinatumomab can induce serial killing of targeted tumor cells since the affinity of blinatumomab for CD3ε subunit (10^−7^ M) is much lower than that for CD19 (10^−9^ M) which increases the mobility of bound T cells^13^. Therefore, T lymphocytes’ effective cell counts and functional activity are two critical factors for the successful outcome of blinatumomab treatment. However, R/R B-ALL patients have been treated with chemotherapy, which has been known to cause a severe reduction of T cell number and function in patients^14–16^. Many relapsed patients were diagnosed after recent bone marrow transplantation, and their T cell number and function were not fully recovered^17^. Consequently, most R/R B-ALL patients are unlikely to respond very well to blinatumomab treatment. Besides, it was reported that a substantial portion of R/R B-ALL patients with a history of or concurrent extramedullary disease failed to respond to blinatumomab, indicating the poor ability of blinatumomab in treating extramedullary disease^18,19^. To improve the treatment efficacy of blinatumomab, we proposed to apply the adoptive transfer of ex vivo expanded γδ T cells to supplement functional T cells in patients.

γδ T cells are a group of unconventional T cells with lytic activity against infected or malignant cells and account for 1–5% of circulating T lymphocytes in humans^20^. γδ T cells express γ and δ heterodimer of T cell receptors (TCR) that recognize non-peptidic phosphoantigens independent of major histocompatibility complex (MHC) restriction and direct intracellular signals through associated CD3 complexes. Like natural killer cells, γδ T cells also express the NKG2D receptor that induces cytolysis upon binding to the stress-inducible proteins such as MHC class I chain-related molecules (MICA, MICB), and UL16-binding proteins (ULBPs) on tumor or stressed cells^21,22^. Based on the expression of the variable (V) region of the TCRδ chain, human γδ T cells can be classified into three main subsets (Vδ1, Vδ2, and Vδ3). Among these subsets, Vδ2 cells almost exclusively coexpress Vγ9 of the TCRγ chain, and Vγ9Vδ2 (also called γ9δ2) T cells represent over 75% of the peripheral γδ T cells^23^. The γ9δ2 T cells expanded ex vivo with aminobisphosphonates have been adoptively transferred into cancer patients, and objective responses were detected without detrimental side effects^24^. In addition, due to the independence of MCH restriction, there are limited risks of graft-versus-host diseases in the allogeneic transfer of γ9δ2 T cells, rendering them attractive for immunotherapy.

This study aims to investigate whether the adoptive transfer of human γ9δ2 T cells can enhance CD19BiTE’s activity against B-cell malignancies. Here we demonstrated that repeated infusion of CD19BiTE and ex vivo expanded human γ9δ2 T cells can prolong survival and attenuate extramedullary disease in mice xenografted with human B-lineage cell line Raji. Our results suggest that the combination of CD19BiTE and the adoptive transfer of human γ9δ2 T cells may be a promising alternative to current immunotherapies for CD19-positive B-cell malignancies.

## Results

### Immunophenotypic profiles and growth curves of expanded human γ9δ2 T cells

**Figure 1:**
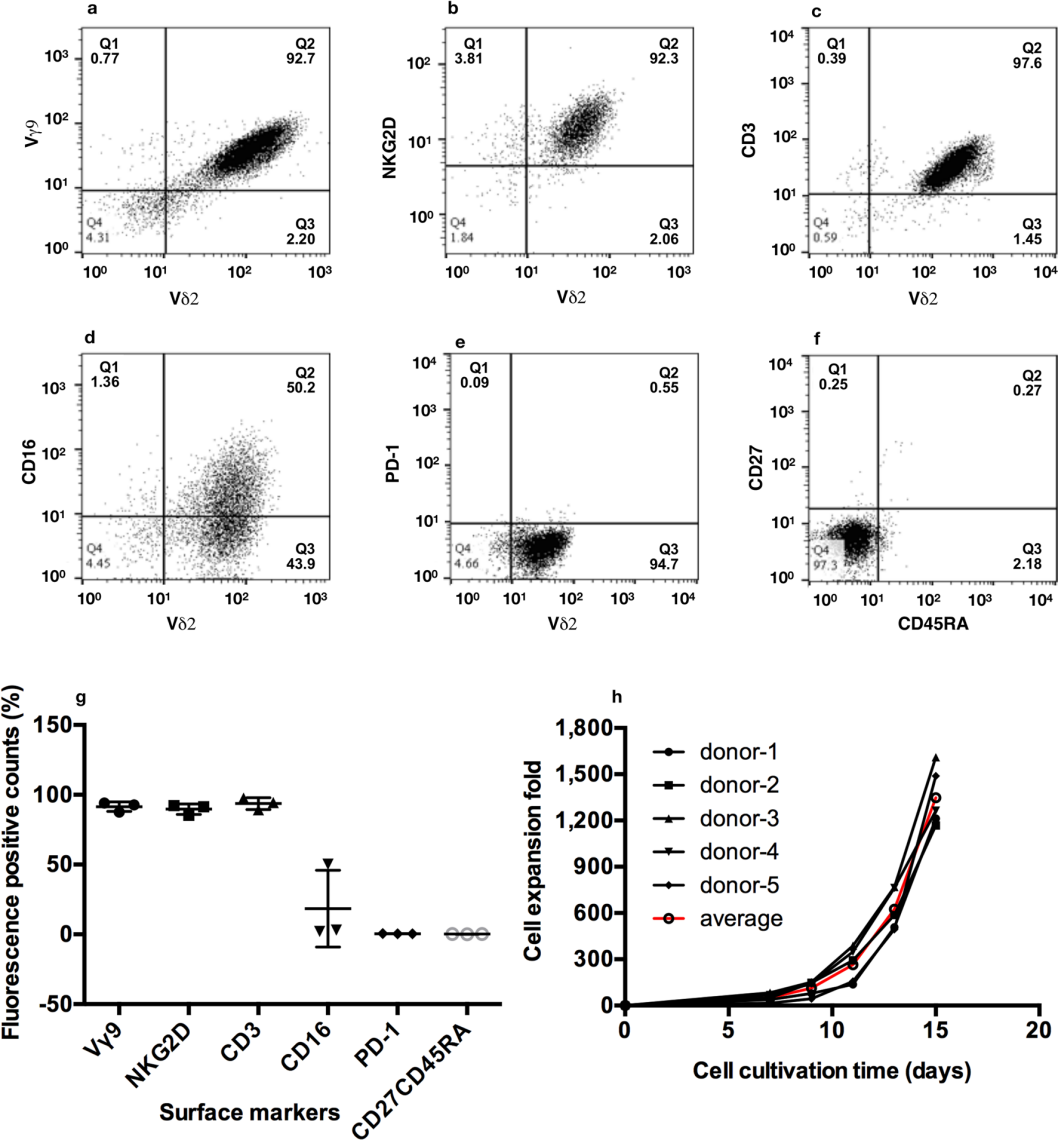
Immunophenotypic profiles of expanded human γ9δ2 T cells. The peripheral blood mononuclear cells (PBMCs) of healthy donors were isolated and grown in the medium formulated for the expansion of γδ T cells for 14 days. The expression of various surface markers was examined by staining with appropriate fluorochrome-labeled monoclonal antibodies (mAbs) and analyzed on a flow cytometer. Images were created using FlowJo software 10.0.0r1. (**a**–**f**) A set of representative surface marker dot plots of a donor’s expanded γ9δ2 T cells. (**a**) Cells were stained with an FITC-labeled mAb against Vγ9 T cell receptor (TCR) and a PE-labeled mAb against Vδ2 TCR to determine the purity of the expanded γδ T cells. The population of γ9δ2 T cells was identified by the expression of Vδ2 TCR and their expression of (**b**) NKG2D, (**c**) CD3, (**d**) CD16, and (**e**) PD-1 was evaluated. (**f**) The expanded γ9δ2 T cells were examined for the expression of CD27 and CD45RA. (**g**) A statistical graph presented the percentage counts of three donors’ expanded γ9δ2 T cells for the expression of various surface markers (Vγ9, NKG2D, CD3, CD16, PD-1, CD27, and CD45RA). Data were expressed as mean ± s.d. (n = 3). (**h**) Growth curve of γ9δ2 T cells. Five donors’ PBMC were cultured for the expansion of γδ T cells for 15 days. At indicated time points (day 0, 7, 9, 11, 15), the total cell number and γ9δ2 percentage were determined by trypan blue staining and flow cytometry analysis, respectively. The calculated cell numbers of expanded γδ T cells were plotted as a line graph with cell expansion fold versus cell cultivation time. Red line (): the average of five curves.

γδ T cells were expanded from the isolated peripheral blood mononuclear cells (PBMC) of healthy donors in the medium with an optimized formulation for the expansion of γδ T cells. It is essential to determine the purity and phenotype of the expanded γδ T cells. On days 14 after culture, cells were stained with appropriate fluorochrome-labeled monoclonal antibodies (mAbs) against the relevant surface markers and subjected to flow cytometry analysis. In a representative donor’s sample, the frequency of γ9δ2 T cells in the expanded culture was more than 92.7% (Fig. 1a). The expression of NKG2D (92.3%; Fig. 1b), CD3 (97.6%; Fig. 1c), and CD16 (50.2%; Fig. 1d) was detected in the high percentage of cultured γ9δ2 T cells, while the expression of PD-1 (0.55%) was hardly detected (Fig. 1e). More than 97% of the cultured cells displayed CD27^−^CD45RA^−^ phenotype, indicating that the dominant population of the expanded γ9δ2 T cells were effector memory T cells (Fig. 1f). Similar expression profiles of those surface markers were found in other two donors’ samples (Vγ9: 91.5 ± 3.4%; NKG2D: 89.7 ± 3.8%; CD3: 93.7 ± 4.2%; PD-1: 0.5 ± 0.1%; CD27^−^CD45RA^−^: 0.2 ± 0.1%; n = 3; Fig. 1g), with the exception of CD16 expression (18.5 ± 27.5%; n = 3; Fig. 1g) (see Supplementary Fig. S1 online). In the other five donors’ samples, γ9δ2 T cell numbers were determined at various time points (day 0, 7, 9, 11, 13, 15) of cell cultivation, and the expansion fold of cell numbers was calculated. The expansion fold gradually increased along with prolong cultivation periods (day 0: onefold; day 7: 50.8 ± 23.6 fold; day 9: 112.8 ± 43.5 fold; day 11: 264.6 ± 101.2 fold; day 13: 624.2 ± 120.1 fold; day 15: 1348 ± 171.3 fold; n = 5; Fig. 1h). These results indicate that the number of γ9δ2 T cells can be effectively expanded from PBMC for more than one thousandfold with purity over 91% within 14–15 days.

### CD19BiTE recognizes CD19^+^ tumor cells and CD3^+^ human γ9δ2 T cells

**Figure 2:**
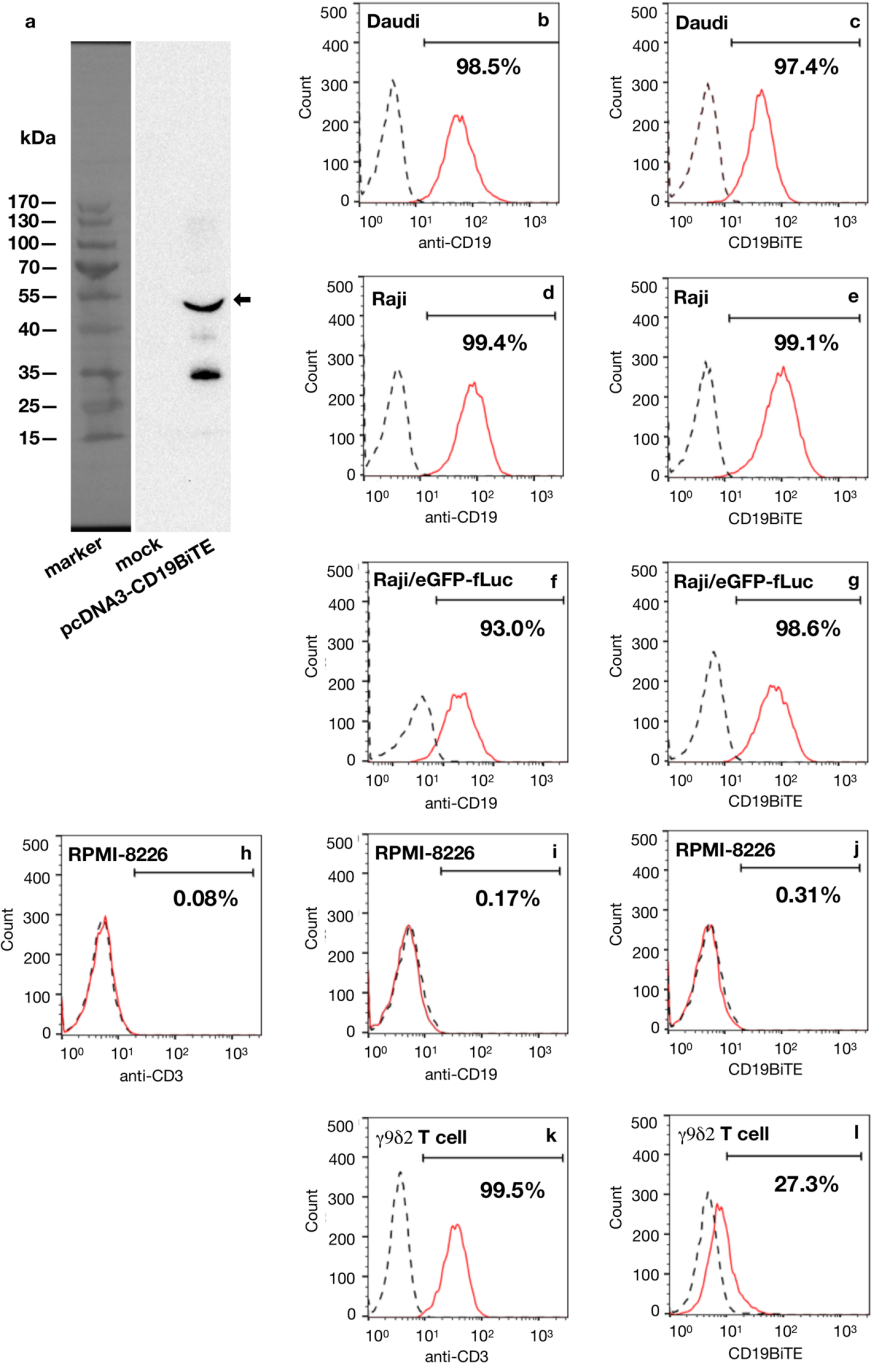
CD19BiTE binds to CD19-positive tumor cells and human γ9δ2 T cells. (**a**) HEK293 cells were transfected without (mock) or with the pcDNA3-CD19BiTE plasmid for 48 h, and then the culture media were subjected to protein purification by Ni-sepharose. The purified products were separated by SDS-PAGE and probed by an anti-6 × -histidine monoclonal antibody (mAb) in the Western blot analysis. A detected major band of the molecular weight of about 55 kDa similar to the predicted size of CD19BiTE was indicated (arrow). (**b**–**l**) Immunofluorescence histograms. Four tumor cell lines, Daudi (**b**), Raji (**d**), Raji/eGFP-fLu (**f**), and RPMI-8226 (**i**), were stained without (dotted black line) or with a PE-labeled anti-CD19 mAb (solid red line). RPMI-8226 (**h**) and expanded human γ9δ2 T cells (**k**) were stained without (dotted black line) or with an FITC-labeled anti-CD3 mAb (solid red line). Daudi (**c**), Raji (**e**), Raji/eGFP-fLu (**g**), and RPMI-8226 (**j**), and expanded human γ9δ2 T cells (**l**) were pre-incubated with (solid red line) or without (dotted black line) CD19BiTE (0.5 ng/µl) and then stained with a PE-labeled anti-6 × -histidine mAb. The stained cells were analyzed on a flow cytometer, and data were presented as histograms with counts versus fluorescence. The percentage of cell populations with fluorescence intensity higher than the background value was indicated. Images were created using FlowJo software 10.0.0r1.

To confirm that the homemade 6 × -histidine-tagged CD19BiTE possesses binding specificity to the cell surface proteins CD19 and CD3, we examined CD19BiTE by Western blot and flow cytometry analyses. The culture media of HEK293 cells transfected without or with the pcDNA3-CD19BiTE plasmid were purified by Ni-sepharose and separated by SDS–polyacrylamide gel electrophoresis (SDS-PAGE) under the reducing condition. By the Western blot analysis, a major band with a molecular weight (55 kDa) similar to the predicted size of CD19BiTE was identified by an anti-6 × -histidine mAb in the transfected but not mock sample. This result demonstrated that 6 × -histidine-tagged CD19BiTE could be generated in the secreted form and purified by Ni-sepharose from the culture medium of HEK293 cells transfected with the pcDNA3-BiTE plasmid (Fig. 2a) (see Supplementary Fig. S2 online). By using fluorochrome-labeled anti-CD19 or anti-CD3 antibodies in the flow cytometry analysis, tumor cell lines Daudi, Raji, and Raji/eGFP-fLuc were shown to be positive for CD19 expression on the cell surface (Fig. 2b, d, f), and the expanded human γ9δ2 T cells were positive for CD3 expression on the cell surface (Fig. 2k); RPMI-8226 was negative for the surface expression of CD3 or CD19 (Fig. 2h, i). When the CD19^+^ tumor cells (Daudi, Raji, and Raji/eGFP-fLuc) and CD3^+^ human γ9δ2 T cells were stained with CD19BiTE + a fluorochrome-labeled anti-6 × -histidine mAb, the mean fluorescence intensity detected by the flow cytometry analysis was higher than that in the respective cells stained with a fluorochrome-labeled anti-6 × -histidine mAb alone (Fig. 2c, e, g, l). No fluorescence difference was found when CD19-negative RPMI-8226 cells were examined under the same staining procedure (Fig. 2j). These results indicate that the homemade CD19BiTE can bind to the cells expressing CD19 and CD3 surface proteins.

### The combination of γ9δ2 T cells and CD19BiTE enhances lysis of CD19^+^ B-lineage cell lines in vitro

**Figure 3:**
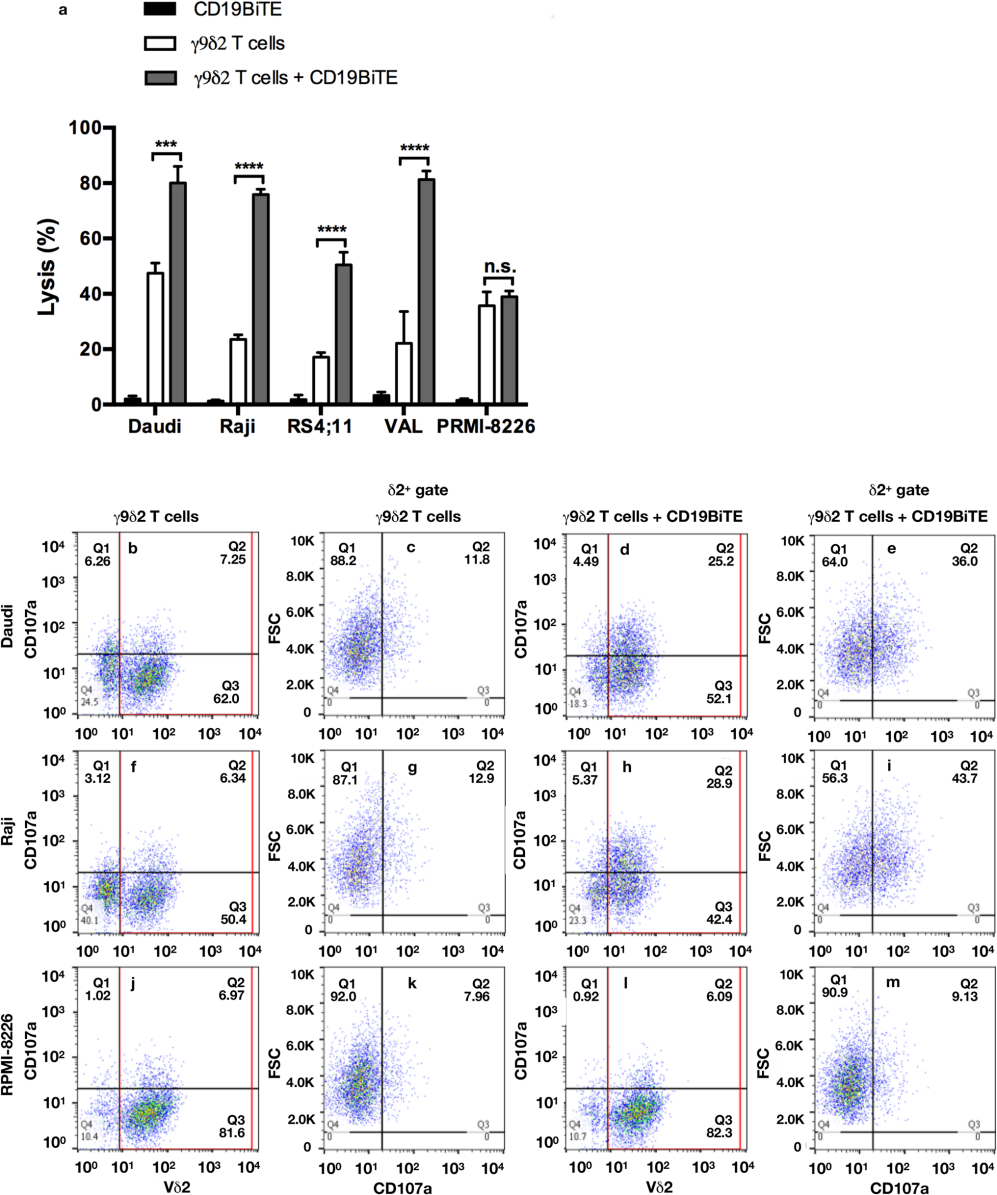
The combination of γ9δ2 T cells and CD19BiTE enhances the lysis of C19^+^ B-lineage cell lines in vitro. (**a**) Cytotoxic assays were conducted at an effector to target ratio (E: T) of 20:1, and the percentage of target cell lysis was determined by flow cytometry analysis. CD19-positive (Daudi, Raji, Rs4;11, VAL) and -negative (RPMI-8226) B-lineage tumor cell lines were labeled with PKH26 and CFSE fluorescence dyes and used as target cells, and the expanded γ9δ2 T cells (derived from three donors) were used as effector cells. Each labeled target cell line (1 × 10^4^) was incubated with CD19BiTE (0.25 ng/µl), γ9δ2 T cells (2 × 10^5^), or γ9δ2 T cells (2 × 10^5^) + CD19BiTE (0.25 ng/µl) at a volume of 100 µl for 5 h, and then its lysis percentage was determined by flow cytometry. Data were plotted as mean ± s.d., and the statistical analysis was performed by one-way analysis of variance (ANOVA) followed by Tukey’s multiple comparison tests. ***: *p* = 0.002, ****: *p* < 0.0001, n.s.: not significant. The replication number (n) in each treatment group was indicated. Black bar (■): tumor cell lines were incubated with CD19BiTE; White bar (□): tumor cell lines were incubated with γ9δ2 T cells; Gray bar (■): tumor cell lines were incubated with γ9δ2 T cells + CD19BiTE. (b ~ m) The cytotoxic activity of a donor’s expanded γ9δ2 T cells was examined by detecting the degranulation marker CD107a. At an effector to target ratio (E: T) of 1:1, three target cell lines (Daudi, Raji, RPMI-8226; 1 × 10^5^) were incubated with γ9δ2 T cells (Daudi: **b**, **c**; Raji: **f**, **g**; RPMI-8226: **j**, **k**), or γ9δ2 T cells + CD19BiTE (0.25 ng/μl) (Daudi: **d**, **e**; Raji: **h**, **i**; RPMI-8226: **l**, **m**) at a volume of 100 µl for 6 h, and then the cell mixtures were subjected to flow cytometry analysis using a PE-labeled anti-CD107a and an FITC-labeled anti-Vγ9 antibodies. On the CD107a versus Vγ9 dot plots (**b**, **f**, **j**, **d**, **h**, **l**), Vγ9 positive counts were gated for evaluating the percentage of CD107a positive cells among Vγ9 positive cells on the forward scatter (FSC) versus CD107a dot plots (**c**, **g**, **k**, **e**, **i**, **m**). Images were created using FlowJo software 10.0.0r1.

Cytotoxicity assays were used to evaluate whether γ9δ2 T cells (derived from three donors) combined with CD19BiTE increases the killing effectivity on CD19^+^ tumor cells (Fig. 3a). The fluorescence-labeled CD19^+^ (Daudi, Raji, RS4;11, VAL) or CD19^−^ (RPMI-8226) B-lineage cell lines were incubated with CD19BiTE, γ9δ2 T cells, or γ9δ2 T cells + CD19BiTE. Five hours after incubation, the lysis percentage of tumor cells were determined by flow cytometry. CD19BiTE did not induce significant lysis, while γ9δ2 T cells significantly increased tumor lysis [Daudi: 47.4 ± 16% (n = 20) vs 2.03 ± 1.9% (n = 3), *p* = 0.0002; Raji: 23.5 ± 19% (n = 120) vs 1.3 ± 1.5% (n = 15), *p* < 0.0001; RS4;11: 17.1 ± 7% (n = 18) vs 1.8 ± 3.2% (n = 4), *p* = 0.0311; VAL: 22.1 ± 11% (n = 25) vs 3.3 ± 2.9% (n = 5), *p* = 0.0025; RPMI-8226: 35.7 ± 12% (n = 6) vs 1.6 ± 0.8% (n = 2), *p* = 0.0127]. This result suggests that γ9δ2 T cells can cause cytotoxicity on various human B-lineage tumor cells, regardless of the expression of CD19 on the target cells. Compared with the treatment with γ9δ2 T cells, treatment with CD19BiTE + γ9δ2 T cells significantly enhanced the lysis percentage of Daudi [80.0 ± 13.5% (n = 5) vs 47.4 ± 16% (n = 20), *p* = 0.0002], Raji [75.9 ± 17.8% (n = 87) vs 23.5 ± 19% (n = 120), *p* < 0.0001], RS4;11 [50.5 ± 15.2% (n = 11) vs 17.1 ± 7% (n = 18), *p* < 0.0001], and VAL [81.3 ± 10.7% (n = 12) vs 22.1 ± 11% (n = 25), *p* < 0.0001], but not RPMI-8226 [39.0 ± 2.9% (n = 2) vs 35.7 ± 12.0% (n = 6), *p* = 0.9237]. This result supports that the combination of γ9δ2 T cells and CD19BiTE enhances lysis of human B-lineage tumor cells in a CD19-restriction manner. We further investigated the T-cell degranulation activity by measuring CD107a expression of a donor’s expanded γ9δ2 T cells (Fig. 3b–m). The CD19^+^ (Daudi, Raji) and CD19^−^ (RPMI-8226) B-lineage cell lines were incubated with γ9δ2 T cells, or γ9δ2 T cells + CD19BiTE. Six hours after incubation, the percentage of CD107a positive counts among γ9δ2 T cells were determined by flow cytometry. γ9δ2 T cells presented higher levels of CD107a in the presence of CD19BiTE than in the absence of CD19BiTE, when they were mixed with Daudi (36% vs. 11.8%; Fig. b–e) or Raji (43.7% vs. 12.9%; Fig. 3f–i), but not with RPMI-8226 (9.13% vs. 7.96%; Fig. 3j–m). This result could support the observation that CD19BiTE enhances γ9δ2 T cells’ cytotoxicity on CD19^+^ B-lineage tumor cells.

### Engrafted human γ9δ2 T cells migrate to various organs in immunodeficient mice

**Figure 4:**
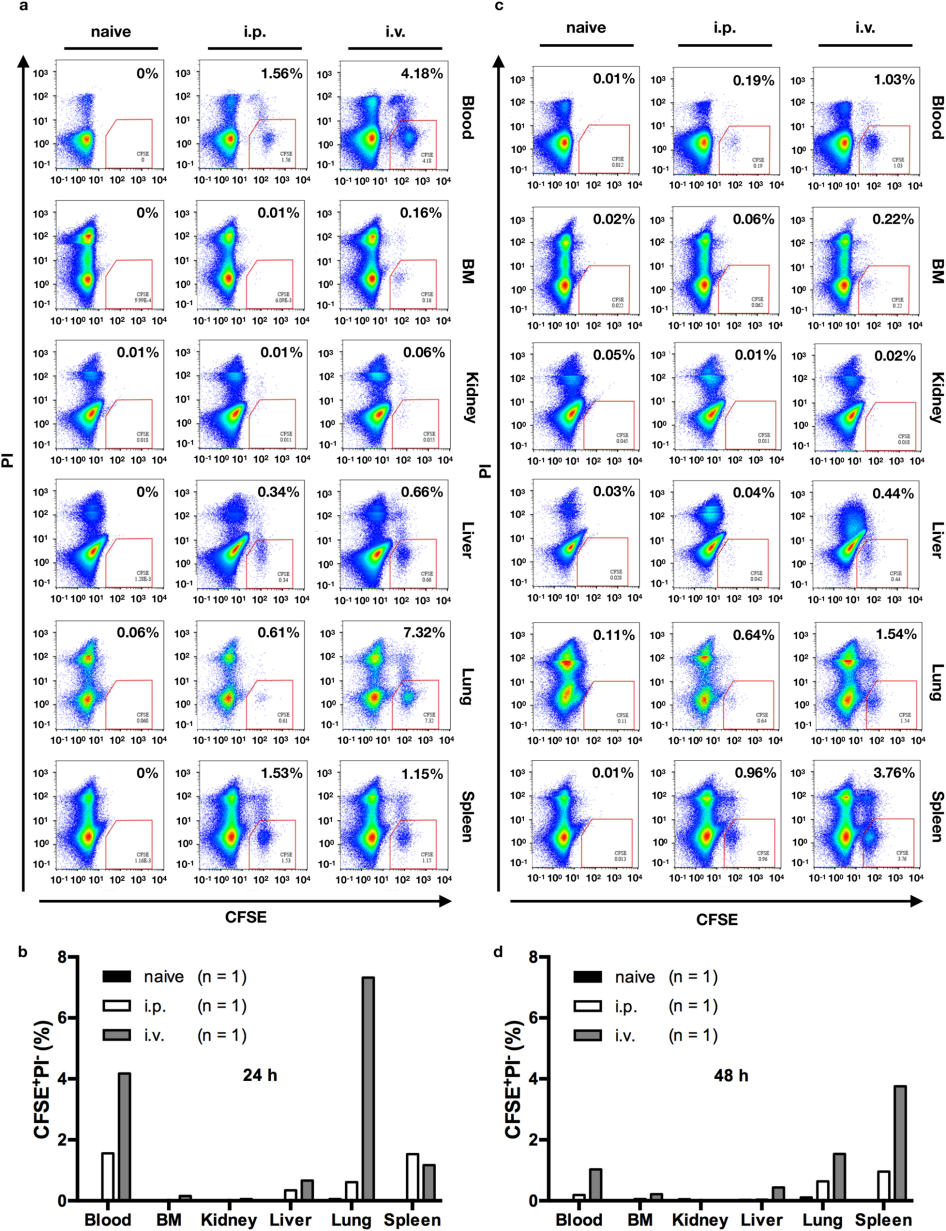
The distribution of engrafted human γ9δ2 T cells in immunodeficient mice. Adult NOG mice (19–20 weeks old, male) were injected with CFSE-labeled human γ9δ2 T cells (derived from one donor; 1.5 × 10^7^ cells/mouse) via the peritoneal cavity (i.p.; n = 2) or lateral tail vein (i.v.; n = 2), or left un-injected (naive; n = 2). At 24 (**a**, **b**) and 48 (**c**, **d**) hours post-injection, animals were sacrificed for the isolation of lymphocytes from the heart blood, femur bone marrow (BM), kidney, liver, lung, and spleen. For each group, one animal was sacrificed at each time point. The isolated cells were stained with propidium iodide (PI), and the percentage of viable γ9δ2 T cells (CFSE^+^PI^−^) was determined by the flow cytometry analysis. Data were presented as dot plots with PI versus CFSE fluorescence (**a**, **c**), and the percentage of CFSE^+^PI^−^ cells in each sample was plotted in bar charts (**b**, **d**). Black bar (■): naive; White bar (□): i.p. injection; Gray bar (■): i.v. injection. Images were created using FlowJo software 10.0.0r1.

To investigate the tissue distribution of engrafted γ9δ2 T cells, we injected CFSE-labeled human γ9δ2 T cells (derived from one donor; 1.5 × 10^7^ cells/mouse) into adult NOG mice through the i.p. or i.v. (via lateral tail veins) route. At 24 and 48 h post-injection, lymphocytes were isolated from various organs and tissues of the injected and non-injected (naive) mice. For each group, one animal was sacrificed at each time point. After propidium iodide (PI) staining, cells were analyzed on a flow cytometer to evaluate the frequency of live engrafted γ9δ2 T cells (CFSE^+^PI^−^) (Fig. 4a, c). No CFSE^+^ cells were detected in all samples from the naive mice, indicating that no auto-fluorescence similar to CFSE fluorescence was emitted in all cell samples. In animals receiving i.p. injection, CFSE^+^PI^−^ cells were detected in the samples derived from the heart blood (24 h: 1.56%, 48 h: 0.19%), liver (24 h: 0.34%, 48 h: 0.04%), lung (24 h: 0.61%, 48 h: 0.64%), and spleen (24 h: 1.53%, 48: 0.96%), but not femur bone marrow (24 h: 0.01%, 48 h: 0.06%), or kidney (24 h: 0.01%, 48 h: 0.01%) (24 h: Fig. 4b; 48 h: Fig. 4d). On the other hand, in animals receiving i.v. injection, CFSE^+^PI^−^ cells were found in the heart blood (24 h: 4.18%, 48 h: 1.03%), femur bone marrow (24 h: 0.16%, 48 h: 0.22%), liver (24 h: 0.66%, 48 h: 0.44%), lung (24 h: 7.32%, 48 h: 1.54%), and spleen (24 h: 1.15%, 48 h: 3.76%); no CFSE^+^PI^−^ cells were found in the kidney (24 h: 0.06%, 48 h: 0.02%) (24 h: Fig. 4b; 48 h: Fig. 4d). In general, a trend of a higher frequency of viable γ9δ2 T cells was found in the mice receiving i.v. injection (except the spleen samples at 24 h post-injection) than in the mice receiving i.p. injection. The sample was too small to allow us to draw a statistical conclusion from that, and further investigation with increased sample size is required to confirm the present observation. However, these results suggest that regardless of the injection routes (i.p. or i.v.), human γ9δ2 T cells can circulate in the peripheral blood and migrate to the femur bone, liver, lung, and spleen, but can hardly migrate to the kidney. The intravenous injection was demonstrated as an efficient route for γ9δ2 T cell administrations in terms of the tissue distribution of engrafted γ9δ2 T cells.

### Adoptive transfer of γ9δ2 T cells enhances CD19BiTE’s therapeutic effect and prolongs the survival of mice receiving CD19^+^ tumor xenograft

**Figure 5:**
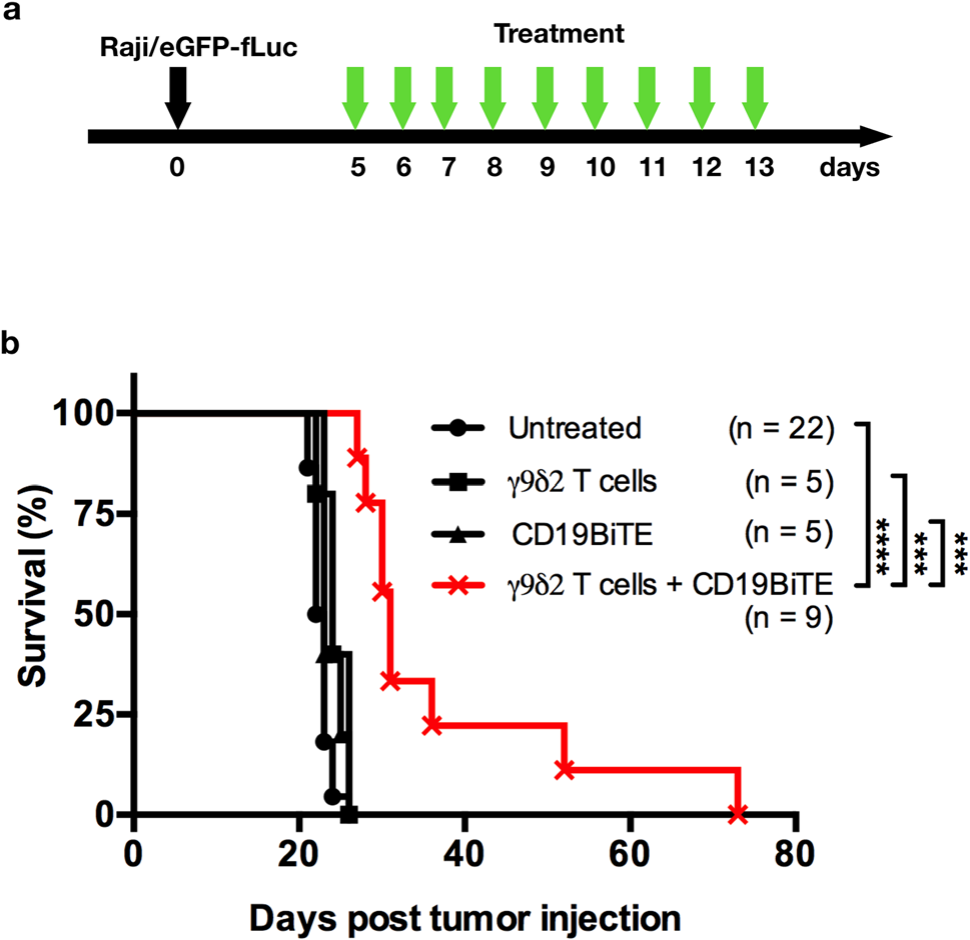
Adoptive transfer of γ9δ2 T cells enhances CD19BiTE’s antitumor activity in CD19^+^ tumor xenograft-bearing mice. (**a**) Schematic representation of the experimental schedule. (**b**) Kaplan–Meier survival curves. Adult NOG mice (16–18 weeks old, male; n = 41) were intravenously (i.v.) inoculated with human Raji/eGFP-fLuc tumor cells (2.5 × 10^5^ cells/mouse). On day five post tumor inoculation, mice received daily treatment of a dose of γ9δ2 T cells (2.5 × 10^6^ cells/mouse, i.v.; n = 5) or two doses of CD19BiTE (250 ng/dose/mouse, i.v.; n = 5) alone, or both (n = 9) for nine consecutive days. Untreated animals serve as controls (n = 22). Survival, the days after tumor inoculation, was followed up. γ9δ2 T cells were expanded from two donors’ peripheral blood mononuclear cells. A single mouse was treated with the γ9δ2 T cells derived from the same donor at different time points. Survival curves were analyzed using the Log-rank (Mantel-Cox) test. ***: *p* = 0.0002; ****: *p* < 0.0001. ●: no treatment; ■: treatment of γ9δ2 T cells; ▲: treatment of CD19BiTE; ✖: combination treatment of γ9δ2 T cells + CD19BiTE.

The therapeutic effect of γ9δ2 T cells and CD19BiTE co-treatment was examined in a Raji-xenografted NOG mouse model (Fig. 5). Raji/eGFP-fLuc tumor cells (2.5 × 10^5^ cells/mouse) were inoculated via tail veins. Five days later, mice were daily injected (i.v.) with one dose of γ9δ2 T cells (n = 5), two doses of CD19BiTE (n = 5), or the combination of both (n = 9) for nine consecutive days, or left untreated as controls (n = 22) (Fig. 5a). γ9δ2 T cells were expanded from two donors’ PBMC. A single mouse was treated with the γ9δ2 T cells derived from the same donor at different time points. Mice receiving γ9δ2 T cells + CD19BiTE co-treatment showed prolonged survival compared with those receiving only γ9δ2 T cells (*p* = 0.0002), CD19BiTE (*p* = 0.0002), or no treatment (*p* < 0.0001) (Fig. 5b). No significant difference was found while the untreated vs. γ9δ2 T cells, untreated vs. CD19BiTE, and γ9δ2 T cells vs. CD19BiTE. This result indicates that the adoptive transfer of γ9δ2 T cells combined with CD19BiTE can display effective antitumor activity against CD19^+^ B-cell malignancy in vivo.

### Adoptive transfer of γ9δ2 T cells in combination with CD19BiTE attenuates extramedullary disease

**Figure 6:**
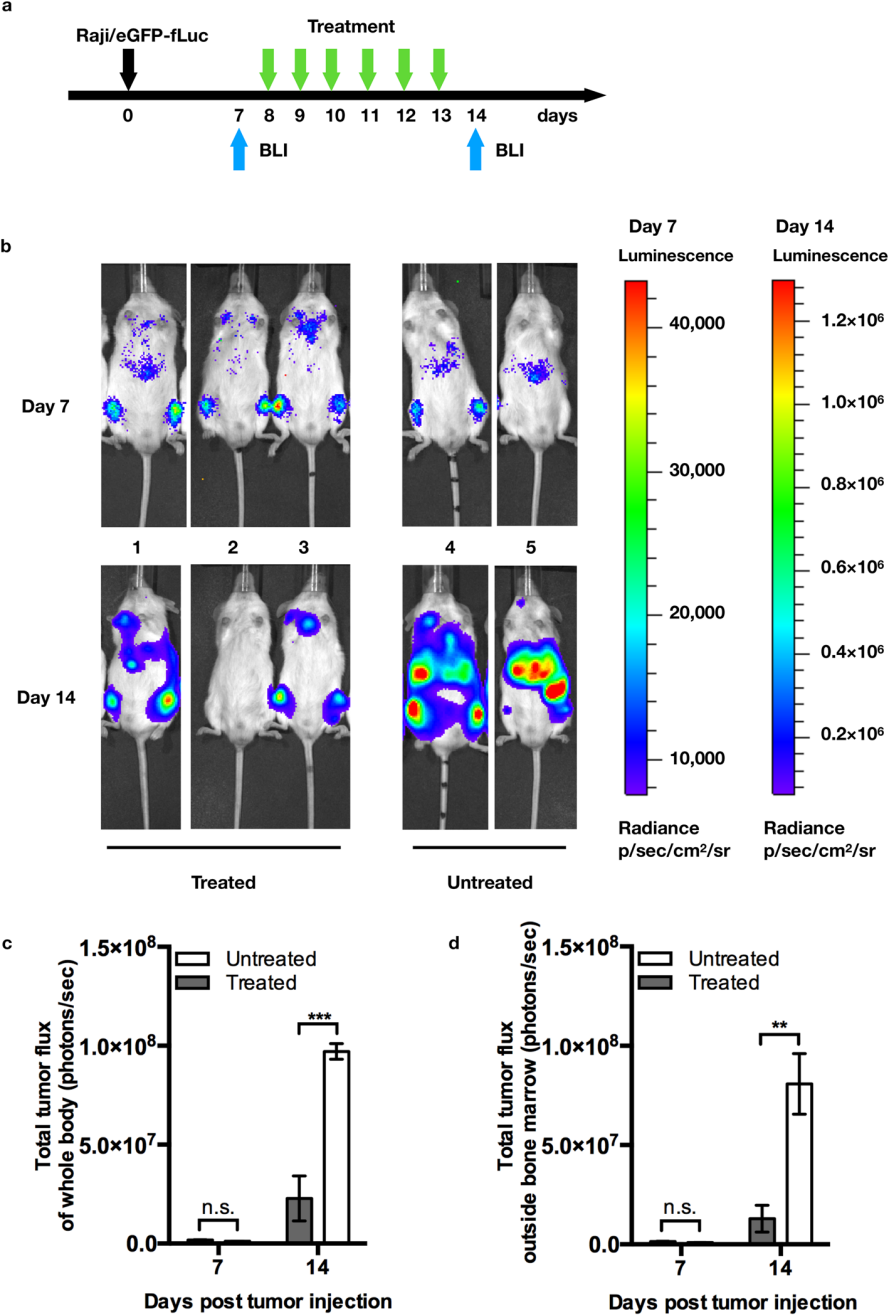
Adoptive transfer of γ9δ2 T cells in combination with CD19BiTE attenuates extramedullary disease. (**a**) Schematic representation of the experimental schedule. Adult NOG mice (40 weeks old, male) were injected with Raji/eGFP-fLuc tumor cells (2.5 × 10^5^ cells/mouse) through the lateral tail vein (i.v.). On day eight post tumor injection, mice received daily treatment of two i.v. doses of CD19BiTE (250 ng/dose/mouse) and an i.v. dose of γ9δ2 T cells (2.5 × 10^6^ cells/mouse) for six consecutive days (n = 3), or left untreated as controls (n = 2). γ9δ2 T cells were expanded from two donors’ peripheral blood mononuclear cells. A single mouse was treated with the γ9δ2 T cells derived from the same donor at different time points. (**b**) Bioluminescence images (BLI) were taken for each mouse on days 7 and 14 after tumor injection. The total flux (photons/sec) of tumor cells in the whole body (**c**) or the area outside the bone marrow (**d**) was calculated as the sum of bioluminescence signal intensity. Data were plotted as mean ± s.d., and the statistical analysis was performed by two-way analysis of variance (ANOVA) followed by Sidak’s multiple comparison tests (**: *p* = 0.0011; ***: *p* = 0.0008; n.s.: not significant).

Acute leukemia can spread to the soft tissues or organs outside the bone marrow and lead to the development of the extramedullary disease that often poses therapeutic dilemmas. As shown in Fig. 5b, only the combination of γ9δ2 T cells and CD19BiTE, but not γ9δ2 T cells or CD19BiTE alone, prolonged survival of tumor-bearing animals. We next examined whether γ9δ2 T cells can attack extramedullary leukemia in the presence of CD19BiTE. Adult NOG mice were injected with Raji/eGFP-fLuc tumor cells (2.5 × 10^5^ cells/mouse, i.v.). On day 8 after injection of tumor cells, mice (n = 3) were treated with γ9δ2 T cells + CD19BiTE (i.v.) daily for six consecutive days, or left untreated as controls (n = 2) (Fig. 6a). γ9δ2 T cells were expanded from two donors’ PBMC. A single mouse was treated with the γ9δ2 T cells derived from the same donor at different time points. The images of whole-body bioluminescence were taken on days 7 and 14 after tumor injection (Fig. 6b) (see Supplementary Fig. S3 online). On day 7, all animals showed similar levels of whole-body bioluminescence intensity. However, on day 14, stronger bioluminescence intensity for the whole body (mean difference: − 7.443 × 10^7^; effect size: − 13.5; 95% CI: − 1.057 × 10^7^ to − 4.318 × 10^7^; *p* = 0.0008) and the area outside the bone marrow (mean difference: − 6.776 × 10^7^; effect size: − 3.1; 95% CI: − 9.772 × 10^7^ to − 3.78 × 10^7^; *p* = 0.0011) was found in the untreated group, compared with the treated group (Fig. 6c, d). Because of the small sample size, the results need to be interpreted with caution. These data indicate that the combination treatment of γ9δ2 T + CD19BiTE ameliorates tumor growth and extramedullary disease in the xenograft mouse model, but further investigation with larger sample size is required to confirm this observation.

## Discussion

In this study, we investigated whether the combination of human γ9δ2 T cells can improve CD19BiTE’s activity against B-cell malignancies in vitro and in vivo. Our data showed that CD19BiTE redirected γ9δ2 T cells to lysis various CD19-positive B-lineage cell lines in the culture condition. In the presence of CD19BiTE, the adoptive transfer of human γ9δ2 T cells significantly prolonged the survival of the immunodeficiency mice engrafted with human CD19-positive B-lineage tumor cells. In addition, the combination of γ9δ2 T cells and CD19BiTE attenuated the development of extramedullary disease in this engrafted mouse model. To the best of our knowledge, these data are the first to illustrate the effectiveness of combinative treatment with the adoptive transfer of γ9δ2 T cells and CD19BiTE for CD19-positive B-lineage cancers.

Consistent with other studies, our ex vivo expanded γ9δ2 T cells also expressed CD16 and NKG2D membrane proteins^25,26^. CD16 (FcγRIIIA) is a receptor for the Fc region of IgG. It can efficiently mediate antibody-dependent cellular cytotoxicity (ADCC) by directing CD16^+^ effector cells to bind to IgG-coated target cells^27^. In addition to the CD19BiTE-mediated cytotoxicity, our cultured γ9δ2 T cells might exhibit ADCC when used in combination with therapeutic monoclonal antibodies against tumors. Several lines of evidence have shown that adoptive transfer of human CD16^+^ γ9δ2 T cells potentiated the in vivo efficacy of antibody drugs—rituximab (anti-CD20 in leukemia) and trastuzumab (anti-HER2 in breast cancer)—in xenograft cancer models^28,29^. On the other hand, most γ9δ2 T cells also express the NKG2D receptor and exert cytotoxicity on the tumor cells expressing MICA/B or ULBPs ligands^26^. Whether our γ9δ2 T cells can act similarly to provide CD16- and NKG2D-mediated actions against tumors is worth evaluating in our future studies.

CD8^+^ αβ T cells can be classified into four subsets based on their expression of CD27 and CD45RA surface markers: naive (CD27^+^CD45RA^+^), central memory (CD27^+^CD45RA^−^), effector memory (CD27^−^CD45RA^−^), and terminally differentiated (CD27^−^CD45RA^+^)^30^. Among all subsets of CD8^+^ T cells, effector memory T cells exert the highest cytotoxicity on tumor cells targeted by CD19BiTE or other BiTE antibodies in the absence of CD28 activation, whereas naive T cells do not show any cytotoxic potential^31^. Like αβ T cells, γ9δ2 T cells can be classified into these four subsets^32^. In accordance with previous studies^28,33,34^, most of our ex vivo expanded γ9δ2 T cells displayed the effector memory phenotype (CD27^−^CD45RA^−^), which might explain that our γ9δ2 T cells exhibited CD19BiTE-mediated killing activity without the requirement of costimulatory signals from CD28.

Programmed cell death-1 (PD-1) is an inhibitory receptor, which is expressed on αβ and γδ T cells after antigenic stimulation on the T cell receptors^35,36^. The interaction of PD-1 with its specific ligands PD-L1 and PD-L2 can diminish T cells’ cytotoxic activity against tumors. It has been reported that most PD-1^+^ human γ9δ2 T cells lose PD-1 expression as ex vivo expansion progresses, but the following stimulation with phosphoantigens no longer leads to re-expression of PD-1^36,37^. Furthermore, the resulting PD-1^−^ human γ9δ2 T cells remain negative for the expression of PD-1 and attenuate tumor growth after transplantation into xenograft mice^37^. In line with previous studies, the dominant population of our expanded human γ9δ2 T cells were PD-1^−^ and showed potent antitumor effects *in vitr*o and in vivo. The lack of PD-1 expression might be an additional advantage of ex vivo expanded γ9δ2 T cells, as the upregulation of PD-L1 on tumors is unlikely to attenuate the cytotoxic function of adoptively transferred γ9δ2 T cells via PD-1/PD-L1 engagement.

In the cytotoxic assay, our ex vivo expanded human γ9δ2 T cells can independently lysis various human B-lineage tumor cells, which may be attributed to the mechanisms related to the antigen recognition via γδ TCR and NKG2D receptors, as demonstrated in other studies^22,38^. In the presence of CD19BiTE, γ9δ2 T cells’ antitumor activity was significantly enhanced in a CD19-restriction manner, indicating that CD19BiTE can redirect γ9δ2 T cells to attack specific target cells through recognition of CD19. Furthermore, treatment with CD19BiTE + γ9δ2 T cells prolonged survival and attenuated extramedullary disease in the mice xenografted with human CD19^+^ B-lineage tumor cells. Our cultured γ9δ2 T cells’ capability to migrate to organs outside the bone marrow (such as lung, liver, and spleen) may partly explain the attenuation of extramedullary disease. These promising in vivo results support the strategy for the treatment of CD19^+^ B-cell malignancies by the combination of CD19BiTE and the adoptive transfer of ex vivo expanded human γ9δ2 T cells. The therapeutic benefits of this strategy still require complete evaluation in clinical trials.

There is currently an expanding list of BiTE antibodies in clinical trials for a range of hematological and solid tumors^39,40^. It is reasonable to envision that the adoptive transfer of human γ9δ2 T cells combined with these candidate BiTEs might be readily applicable to various malignancies in the near future. However, more works are required to translate our observation into clinical benefits. Unlike CAR-T immunotherapy, which requires tedious steps to modify patients’ T cells with chimeric-antigen receptor genes to target the cancer cells, it is relatively easy and cost-effective for clinical practice that BiTE directs γ9δ2 T cells to the target without the genetic modification.

There are some limitations to the present study. In the in vitro and in vivo experiments, human B-lineage tumor cell lines were used as target cells, and γ9δ2 T cells expanded from the PBMC of healthy donors were used as effector cells. This experimental setting did not fully reflect the clinical situation that the target and effector cells are derived from the same patients. Therefore, the results should be interpreted with caution. Although we have demonstrated the antitumor effect of γ9δ2 T cells + CD19BiTE in vivo in comparison with that of γ9δ2 T cells or CD19BiTE alone, another control with PBMC + CD19BiTE will further highlight whether there is a potential advantage of using γ9δ2 T cells + CD19BiTE.

In summary, the adoptive transfer of ex vivo expanded human γ9δ2 T cells combined with CD19BiTE may provide an efficient therapy for CD19-positive B-cell cancers. Co-administration of γ9δ2 T cells and specific BiTE molecules may also be applied as a new strategy for treating other malignancies.

## Methods

### Cell lines

The human embryonic kidney cell line HEK293 (#60019, Bioresource Collection and Research Center [BCRC], Taiwan), used for the production of recombinant lentivirus vectors and CD19BiTE, was maintained in Dulbecco’s modified Eagle’s medium (DMEM) containing 10% heat-inactivated fetal bovine serum (FBS; #SH30071.03, Hyclone), penicillin (100 IU/ml), and streptomycin (100 µg/ml). Four CD19-positive human B-lineage cell lines, Daudi (#60192, BCRC, Taiwan), Raji (#60116, BCRC, Taiwan), RS4;11 (#ACC-508, DSMZ, Germany) and VAL (#ACC-586, DSMZ, Germany), and a CD19-negative human B-lineage cell line, RPMI-8226 (#60384, BCRC, Taiwan) were maintained in RPMI-1640 medium supplemented with 10% heat-inactivated FBS, penicillin (100 IU/ml), and streptomycin (100 µg/ml). All cell lines were grown at 37 °C in a humidified incubator with 5% CO_2_. Unless otherwise indicated, all cell culture reagents were purchased from Invitrogen Corporation.

### Ex vivo expansion of human γ9δ2 T cells

Written informed consent was obtained from every voluntary healthy donor before the collection of peripheral blood samples. This study was conducted in accordance with the protocol (FJU-IRB: C107013) approved by the Institutional Research Board of Fu Jen Catholic University, Taiwan. All methods were carried out in accordance with relevant guidelines and regulations. Peripheral blood mononuclear cells (PBMC) were isolated from 10 ml venous blood by density gradient centrifugation using Ficoll-Paque PLUS (#GE17-1440-02, Sigma) according to the manufacturer’s instruction. For the expansion of γ9δ2 T cells, PBMC (2 × 10^6^ cells/ml) were stimulated with recombinant human IL2 (25 ng/ml; #CYT-209, Prospec) and Zoledronate (1 µM; #1724827, Sigma) for 14–15 days in RPMI-1640 medium containing 10% heat-inactivated FBS, penicillin (100 IU/ml), and streptomycin (100 µg/ml) at 37 °C in a humidified incubator with 5% CO_2_.

### Animals

The immunodeficient NOG mice (NOD/Shi-scid/IL2γ^null^) were used in this study. Mice were group-housed in the cages with individual ventilation under environmentally controlled conditions (12-h light–dark cycle with lights on at 7:00 a.m., 24 ± 2 °C room temperature, 50 ± 10% relative humidity). They had free access to autoclaved food and drinking water (pH 4.0). All animals had an acclimation period of 2 weeks after arrival at the experiment location. All animal experiments were conducted in strict accordance with the animal protocol (Permit Number: NHRI-IACUC-107160-A) reviewed and approved by the Institutional Animal Care and Use Committee at the National Health Research Institutes in Taiwan. All methods were carried out in accordance with ARRIVE guidelines.

### Generation of CD19BiTE

The nucleic acid sequence of CD19BiTE was extracted from patent US7635472B2. The DNA of CD19BiTE was synthesized by GeneDireX Inc. and then subcloned into the expression vector pcDNA3 (Invitrogen) at HindIII and ApaI restriction sites. The resulting construct pcDNA3-CD19BiTE was transformed into *E. coli* TOP10 (#C404010, Invitrogen) for amplification and then purified on a large scale using the plasmid purification kit (#12381, Qiagen). To produce CD19BiTE proteins, we transfected HEK293 cells (80% confluent in 150-mm dishes) with pcDNA3-CD19BiTE (25 ng/dish) using jetPEI transfection reagents (#101-10N, Polyplus). 48 h after transfection, the culture medium was collected for the purification of CD19BiTE by Ni-sepharose (#17-3712-01, GE), according to the manufacturer’s instructions. The purified CD19BiTE was concentrated by spin columns with 30 K molecular weight cut off (MWCO) (#28-9323-61, GE), sterilized by filtering through a 0.2 μm pore size membrane filter, and then stored at − 80 °C until use.

### Western blot

Western blot analysis was performed as described previously^41,42^. The purified CD19BiTE was reconstituted in sterile PBS at a concentration of 5 ng/µl, and a portion of the reconstitution was mixed with an equal volume (25 µl) of 2 × Laemmli buffer (4% SDS, 125 mM Tris–HCl pH6.8, 10% β-mercaptoethanol, 20% glycerol, and 0.004% bromophenol blue in distilled water). After heating at 90 °C for 10 min, 15 ng of CD19BiTE was subjected to electrophoresis separation on a 12% SDS–polyacrylamide gel. The the separated proteins were electrophoretically transferred from the gel to a PVDF membrane (#RPN303F, GE Healthcare). The membrane was incubated with blocking buffer (5% skim milk and 0.1% Tween-20 in PBS) for 1 h and then incubated with a mouse monoclonal antibody against the 6 × -histidine tag (1:3,000; #GTX628914, GeneTex) in 10 ml blocking buffer for 2 h with gentle agitation. After washing with 0.1% Tween-20 (in PBS) three times for 10 min each, the membrane was incubated with a horseradish peroxidase (HRP)-conjugated goat polyclonal antibody (1:10,000; #GTX213111-01, GeneTex) against mouse IgG in 10 ml blocking buffer for 1 h, followed by the washing procedure as described above. The light emission signals of the target proteins on the membrane were generated using an enhanced chemiluminescence reagent (#RPN2106, GE Healthcare) and captured by the CCD camera-based image system MultiGel-21 (TOPBIO, Taiwan).

### Flow cytometry analysis

#### Phenotyping of γδ T cells

The expanded γδ T cells (2 × 10^5^) were pelleted by centrifugation at 400 × *g* for 5 min, followed by resuspension in 40 µl of PBS. Two appropriate fluorochrome-labeled monoclonal antibodies (mAbs; each 5 µl) were added to stain the relevant surface markers. After incubation at 4 °C for 1 h, the stained cells were washed once with PBS and analyzed on a flow cytometer (#0500-5009, Guava easyCyte 5 HPL/guavaSoft software version 2.1, Millipore) where at least 10,000 events were acquired per sample. The collected data were analyzed with FlowJo software (version 10.0.8r1; Attached Dongle ID: AA04012700018517) and presented as dot plots with quadrant gates. The following mAbs were used: anti-Vγ9 FITC (#TCR1720, Invitrogen), anti-Vδ2 PE (#555739, BD), anti-Vδ2 FITC (#562088, BD), anti-CD3 FITC (#555332, BD), anti-CD16 PE (#555407, BD), anti-CD27 PE (#560985, BD), anti-CD45RA FITC (#561882, BD), anti-NKG2D PE (#561815, BD), and anti-PD1 PE (#560908, BD).

#### CD19BiTE binding

For detecting the expression of CD19 and CD3, Daudi, Raji, Raji/eGFP-fLu, and RPMI-8226 cell lines (2 × 10^5^ cells/reaction), and γ9δ2 T cells (2 × 10^5^ cells/reaction) were stained with 2 µl of a PE-labeled anti-CD19 antibody (1:50; #130113731, Miltenyi Biotech) or an FITC-labeled anti-CD3 antibody (1:50; #555332, BD) in 100 µl of PBS at 4 °C for 1 h. After washing once with PBS, cells were subjected to flow cytometry analysis, and the data were expressed as a histogram with counts versus fluorescence. For evaluating CD19BiTE binding, Daudi, Raji, Raji/eGFP-fLu, RPMI-8226, and γ9δ2 T cells (2 × 10^5^ cells/reaction) were incubated with 50 ng CD19BiTE in 100 µl of PBS at 4 °C for 1 h. Cells were then pelleted by centrifugation at 400 × *g* for 5 min, washed once with PBS, and stained with 2 µl of a PE-labeled anti-6 × -histidine tag antibody (1:50; #130098810, Miltenyi Biotech) in 100 µl of PBS at 4 °C for 1 h. Finally, cells were washed once with PBS and subjected to flow cytometry analysis, and the data were expressed as a histogram with counts versus fluorescence. The acquired data were analyzed with FlowJo software (version 10.0.8r1; Attached Dongle ID: AA04012700018517).

### Cytotoxic assay

The cytotoxic function of effector cells was investigated using the fluorometric assessment of T lymphocyte antigen-specific lysis (FATAL) assay^43^. PKH26 is a highly fluorescent lipophilic dye that can stably stain the cell membrane without disturbing surface marker expression. It has been widely used for in vitro and in vivo cell tracking studies. The target cells labeled by PKH26 can be easily identified by flow cytometry. CFDA-SE is a cell-permeable dye that can passively diffuse into cells. Once inside the cell, it is converted by intracellular esterases to a fluorescent product (CFSE) covalently coupled to intracellular proteins and retained within the cell but not transferred to adjacent cells. When cytolysis occurs, CFSE labeled cells lost fluorescent dye-protein conjugates that are not cell-permeable or taken up by live cells. Therefore, the reduction rate of CFSE + cells among the PKH26 + target cells represents the strength of cytotoxic activity. According to the manufacturer’s instructions, target cells were labeled with PKH26 (#SI-MINI26, Sigma) and CFSE (#C34554, Invitrogen) at room temperature. Briefly, cells (2 × 10^6^) were suspended in 50 µl of Diluent-C solution and mixed with 50 µl of 5 µM PKH26 (in Diluent-C) for 6 min, followed by incubation with 100 µl of heat-inactivated FBS for 1 min. Cells were then pelleted by centrifugation at 400 × *g* for 5 min, washed once with 1 ml of PBS, and suspended in 400 µl of PBS. The suspended cells were mixed thoroughly with 1 µl of 50 µM CFSE by pipetting for 5 s, followed by incubation with 400 µl of heat-inactivated FBS for 1 min. Cells were then washed once as described above and suspended in 500 µl of RPMI-1640 medium, and finally, the cell density was determined by trypan blue staining. For the cytotoxic assay, 1 × 10^4^ labeled target cells were incubated with 2 × 10^5^ effector cells (expanded γ9δ2 T cells; derived from three donors) in 100 µl of RPMI-1640 medium with or without CD19BiTE (0.25 ng/µl) in a humidified 5% CO_2_ incubator at 37 °C for 5 h. After that, the total contents were mixed with 120 µl of PBS and then subjected to flow cytometry analysis on the EasyCyte™ 5 Base System instrument (Millipore). Upon excitation at 488 nm, the fluorescence emission of two fluorochromes was collected at 530 nm for CFSE and 586 nm for PKH26. The acquired data were analyzed with FlowJo software (version 10.0.8r1; Attached Dongle ID: AA04012700018517). A forward scatter versus side scatter (FSC/SSC) dot plot was created, and a region of interest was drawn to exclude the debris population. In the chosen region, cells were gated on the PKH26^+^ events before gating on CFSE^+^ events in a histogram of counts versus fluorescence. The following formula calculated the percent killing: [1 − (mean fluorescence intensity of CFSE in the experimental group × counts)/(mean fluorescence intensity of CFSE in the control group × counts)] × 100%.

### CD107a detection assay

Target cells (1 × 10^5^) were mixed with expanded γ9δ2 T cells (1 × 10^5^) in 100 μl of RPMI-1640 medium with or without CD19BiTE (0.25 ng/µl) and supplemented with 2 µl of PE-labeled anti-CD107a antibodies (clone H4A3, #555801, BD). The mixture was loaded in a 96-well plate and incubated in a humidified 5% CO_2_ incubator at 37 °C for 1 h, followed by the addition of 1 µl of the GolgiStop protein transport inhibitor reagent (#554724, BD) and incubation for 4 h. Next, 1 µl of an FITC-labeled anti-Vδ2 antibody (#562088, BD) was added, and the mixture was incubated for 1 h. Finally, cells were transferred to 1.5 ml micro-tubes, pelleted by centrifugation at 400 × *g* for 5 min, washed once with 1 ml of PBS, and suspended in 500 µl of RPMI-1640 medium for flow cytometry analysis. The acquired data were analyzed with FlowJo software (version 10.0.8r1; Attached Dongle ID: AA04012700018517).

### Genetic modification of Raji cells for the expression of eGFP and fLuc

For constructing the bicistronic lentiviral transfer plasmid pLV-eGFP-fLuc, the IVS-IRES sequence and the firefly luciferase (fLuc) gene were amplified from plasmids pIRESpuro3 (#631619, Clontech) and pCMV-GFP-T2A-Luciferase (#BLIV101PA, System Biosciences), respectively, and subcloned into the self-inactivating lentiviral transfer plasmid pHR-SIN-CSGW^44^. The pseudotyped lentivirus vector encoding eGFP and fLuc was generated by cotransfection of pLV-eGFP-fLuc (4.5 µg), pCMVR8.91 (3 µg; encoding lentivirus packaging proteins), and pMD-G (3 µg; encoding VSV-G membrane protein) into HEK293 cells in a 75-T flask using jetPEI transfection reagents (#101-10N, Polyplus)^44^. At 48 h post-transfection, the culture medium (10 ml) containing the lentiviruses was harvested, filtered through 0.2 µm filters, aliquoted, and stored at − 80 °C until use. The cell line Raji/eGFP-fLuc expressing both eGFP and fLuc was generated by lentivirus transduction. Briefly, Raji cells (1 × 10^6^) were pelleted, suspended in 1 ml of the harvested lentivirus medium supplemented with protamine sulfate (10 µg/ml), and centrifuged at 600 × *g* at room temperature for 90 min. After that, cells were grown in RPMI-1640 medium supplemented with 10% heat-inactivated FBS for 5 days. The cells positive for high levels of eGFP were purified by the fluorescence-activated cell sorting (FACS) technique and expanded for further use.

### Tissue distribution of engrafted γ9δ2 T cells

The expanded human γ9δ2 T cells (derived from one donor; 8 × 10^7^) were pelleted by centrifugation at 400 × *g* for 5 min, washed once with 1 ml of PBS, and suspended in 400 µl of PBS. The suspended cells were mixed thoroughly with 4 µl of 50 µM CFSE by pipetting and then left at room temperature for 5 min, followed by incubation with 400 µl of heat-inactivated FBS for 1 min. Cells were then washed once as described above and suspended in 500 µl of PBS for trypan blue staining to determine the cell density. Finally, the cell density was adjusted to 1.5 × 10^7^ cells/200 µl in PBS. Six adult NOG mice (19–20 weeks old, male) were randomly divided into three groups (n = 2 for each group) and then injected with CFSE-labeled cells (1.5 × 10^7^ cells/200 µl/mouse) through the peritoneal cavity or lateral tail veins, or left un-injected as controls. At 24 and 48 h post-injection, mice were sacrificed, and lymphocytes were isolated from the heart blood, femur bone marrow, kidney, liver, lung, and spleen. For each group, one animal was sacrificed at each time point. The harvested cells were stained with propidium iodide (PI; 1 µg/ml) and then analyzed on a flow cytometer to determine the percentage of live engrafted γ9δ2 T cells (CFSE^+^PI^−^). The acquired data were analyzed with FlowJo software (version 10.0.8r1; Attached Dongle ID: AA04012700018517).

### Mouse xenograft studies

#### Survival rate

NOG mice (16–18 weeks old, male; n = 41) were inoculated with 2.5 × 10^5^ Raji/eGFP-fLuc cancer cells (200 µl in PBS) via lateral tail veins (i.v.). On day 5 post-inoculation of cancer cells, mice were randomly divided into four groups and i.v. injected daily with CD19BiTE (n = 5; 250 ng/50 µl/dose/mouse, two doses per day with a 5-h interval), γ9δ2 T cells (n = 5; 2.5 × 10^6^/100 µl/mouse/day), or CD19BiTE + γ9δ2 T cells (n = 9), or left untreated (n = 22) for nine consecutive days. In the group administered with CD19BiTE + γ9δ2 T cells, CD19BiTE (250 ng/50 µl/dose/mouse, two doses per day) was injected at 30 min before and 4.5 h after injections of γ9δ2 T cells (2.5 × 10^6^/100 µl/mouse/day). γ9δ2 T cells were expanded from the PBMC isolated from two donors. In each treated mouse, the γ9δ2 T cells of nine consecutive infusions were derived from the same donor. Mice were evaluated for health status once per day and humanely euthanized when they become moribund or lost > 20% of body weight. Survival times, defined as days after inoculation of cancer cells, were plotted in Kaplan–Meier curves and statistically analyzed with the Log-rank test. On day 5, after transplantation of tumor cells, if the animals did not show signs of severe pain or distress, they were included, but if they did, they were excluded; there was no exclusion in this experiment. In this experiment, we planned to treat 10 animals with CD19BiTE, 10 animals with γ9δ2 T cells, and 11 animals with γ9δ2 T cells + CD19BiTE, and left 10 animals untreated. However, on the first day of treatment, we only had enough materials to treat 5 animals with CD19BiTE, 5 animals with γ9δ2 T cells, and 9 animals with γ9δ2 T cells + CD19BiTE. Therefore, we end up having 22 animals untreated as a control. The random numbers were generated using the RAND function in *Apple’s Numbers* to allocate mice to experimental groups. The investigator who was conducting the treatment could not be blinded to allocation groups. The animal care staff, the person collecting experimental measurements, and the person analyzing the data were unaware of which specific treatment each group received.

#### Extramedullary disease

On day 0, NOG mice (40 weeks old, male) were inoculated with 2.5 × 10^5^ Raji/eGFP-fLuc cancer cells (200 µl in PBS) via lateral tail veins (i.v.). On day 7, mice were anesthetized with 4% isoflurane and injected with D-luciferin (200 mg/kg, i.p.; #L8220, BIOSYNTH Carbosynth) for evaluating tumor growth and distribution using the IVIS in vivo image system (Xenogen). On day 8, animals with similar density and distribution of bioluminescence were randomly divided into two groups and received daily treatment of CD19BiTE + γ9δ2 T cells for six consecutive days (n = 3), or left untreated (n = 2). CD19BiTE (250 ng/50 µl/dose/mouse, two doses per day, i.v.) was injected at 30 min before and 4.5 h after injection of γ9δ2 T cells (2.5 × 10^6^/100 µl/mouse/day, i.v.). γ9δ2 T cells were expanded from the PBMC isolated from two donors. In each treated mouse, the γ9δ2 T cells of six consecutive infusions were derived from the same donor. On day 14, bioluminescence images were taken, as described above, to compare the tumor growth and distribution between groups. The original IVIS images were provided as supplementary materials. In this experiment, the untreated animals became moribund on day 14 post tumor inoculation. Thus, after taking the IVIS images, we euthanized all animals and terminated the experiment in accordance with the approved animal protocol (NHRI-IACUC-107160-A). Therefore, in this experiment, we did not follow up on the survival of all animals.

### Statistical analysis

Data were expressed as mean ± standard deviation (s.d.) and analyzed by using GraphPad Prism 6.0 statistical program (GraphPad Software, Inc). The data normality was examined by D’Agostino & Pearson omnibus normality test, and the normality assumption was not violated. In the in vitro cytotoxic assay, the comparison of lysis percentage among three groups was analyzed by one-way analysis of variance (ANOVA) followed by Tukey’s post-test. In the in vivo study, the comparison of tumor growth between groups at different time points was performed by two-way ANOVA followed by Sidak’s multiple comparison tests. The difference in survival between groups was analyzed using the Log-rank (Mantel-Cox) test. The difference was considered statistically significant when the two-tailed *p*-value was less than 0.05.

## Supplementary Information

Supplementary Information 1.
