# Supplementary material for: The potential of adoptive transfer of γ9δ2 T cells to enhance blinatumomab’s antitumor activity against B-cell malignancy
Source: Sci Rep. 2021 Jun 11;11:12398. doi: 10.1038/s41598-021-91784-1 (PMC8195997; doi:10.1038/s41598-021-91784-1)
Supplement: Supplementary file 1 — Supplementary Information 1. [file 41598_2021_91784_MOESM1_ESM.pdf]

The potential of adoptive transfer of γ9δ2 T cells to enhance blinatumomab's antitumor activity against B-cell malignancy

Yun-Hsiang Chen, Yun Wang, Cheng-Hao Liao, Shu-Ching Hsu

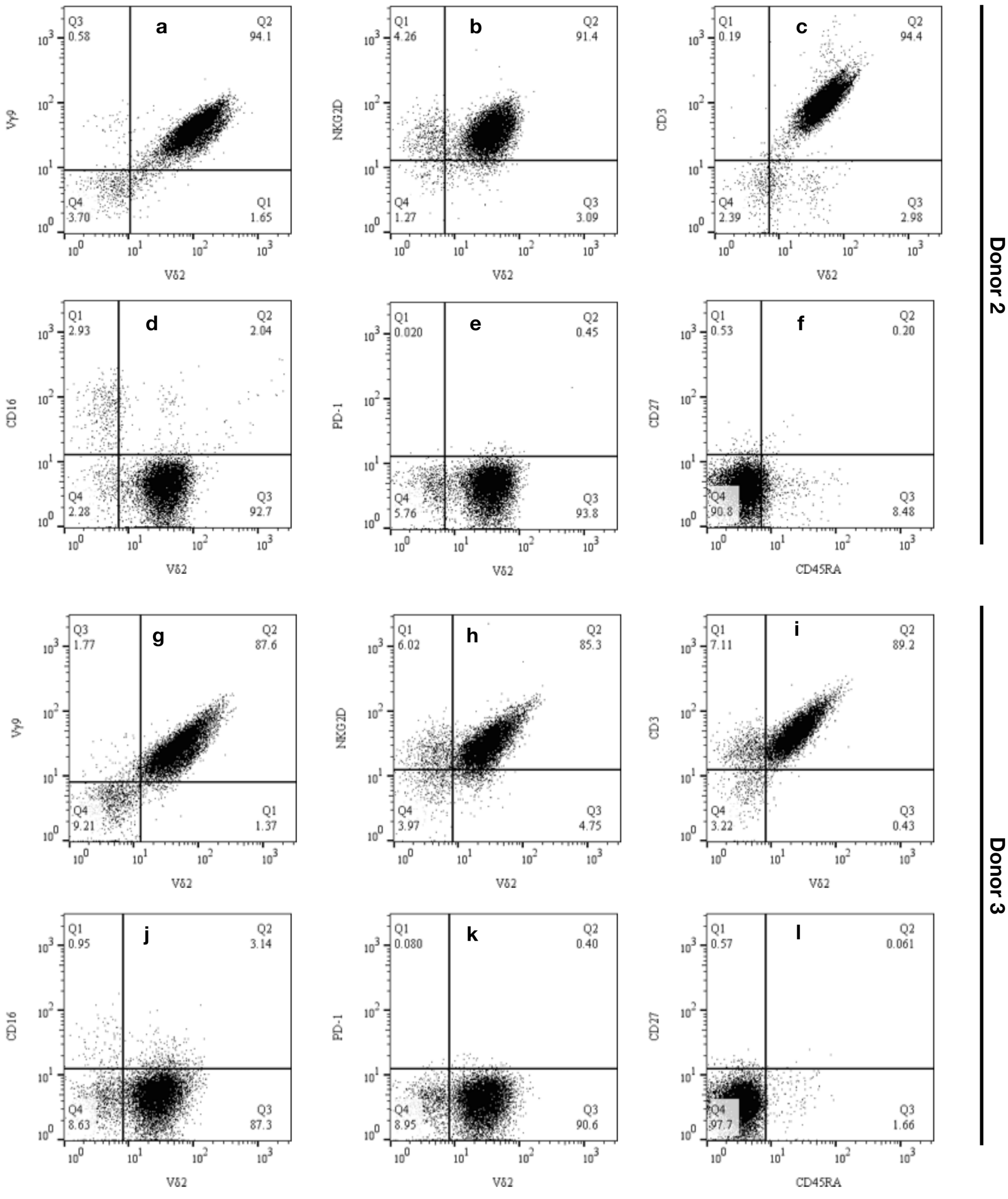

Fig. S1. Immunophenotypic profiles of expanded human γ9δ2 T cells. The expression of various surface markers (Vγ9, NKG2D, CD3, CD16, PD-1, CD27, and CD45RA) on expanded γ9δ2 T cells was examined by flow cytometry analysis. The data collected from donor-1 (Fig. 1a ~ 1f), donor-2 (a ~ f) and donor-3 (g ~ l) were plotted in the statistical graph Fig. 1g.

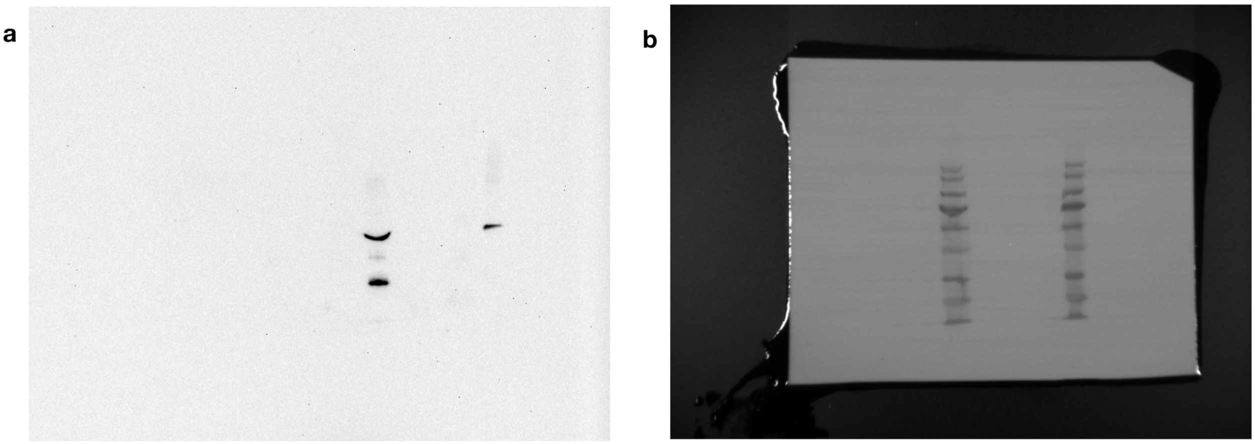

Fig. S2. Detection of the purified CD19BiTE by Western blot analysis. These original unprocessed images (a, b) of Western blot analysis were cropped and presented in Fig. 2a. (a) The light emission signals of the purified CD19BiTE on the PVDF membrane were generated using an enhanced chemiluminescence reagent and captured by the CCD camera-based image system MultiGel-21. (b) The image of the same PVDF membrane was taken under visible light. These two images were cropped and merged, as shown in Fig. 2a.

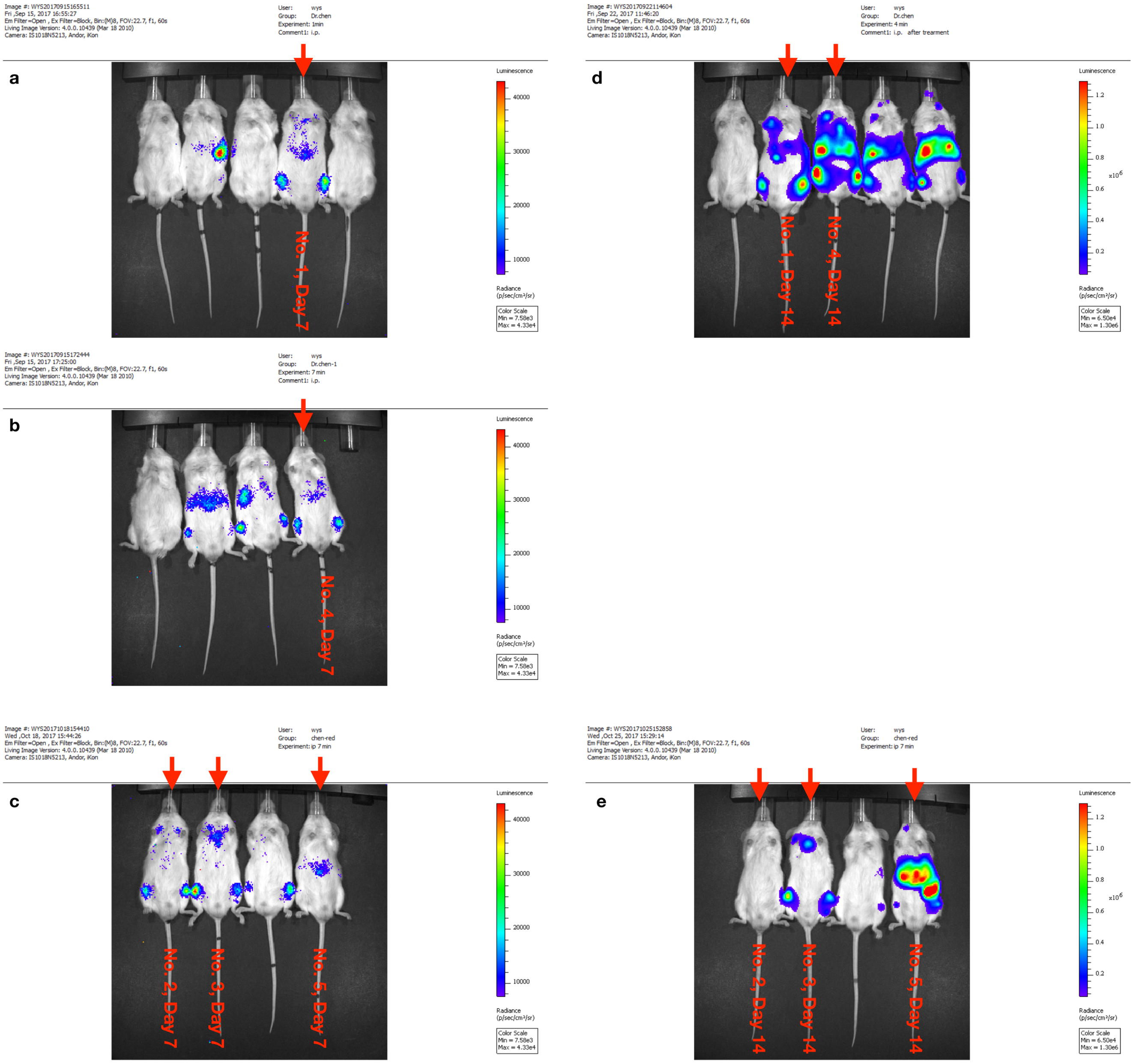

Fig. S3. Therapeutic effects of $\gamma 9\delta 2$ T cells combined with CD19BiTE on the extramedullary disease. These original unprocessed images of the bioluminescence images (a ~ e) were cropped and presented in Fig. 6b. (a) The image of the arrow-indicated animal was cropped and presented as the number-1 animal of day 7 post tumor transplantation in Fig. 6b. (b) The image of the arrow-indicated animal was cropped and presented as the number-4 animal of day 7 post tumor transplantation in Fig. 6b. (c) The images of the arrow-indicated animals were cropped and presented as number-2, -3, and -5 animals of day 7 post tumor transplantation in Fig. 6b. (d) The images of the arrow-indicated animals were cropped and presented as number-1 and -4 animals of day 14 post tumor transplantation in Fig. 6b. (e) The images of the arrow-indicated animals were cropped and presented as number-2, 3, and -5 animals of day 14 post tumor transplantation in Fig. 6b.
